# Supplementary material for: Latent transition analysis of cardiac arrest patients treated in the intensive care unit
Source: PLoS One. 2021 May 27;16(5):e0252318. doi: 10.1371/journal.pone.0252318 (PMC8158944; doi:10.1371/journal.pone.0252318)
Supplement: Attachment — Submitted filename: rebuttal letter.docx [file pone.0252318.s001.docx]

To Dr. Saraschandra Vallabhajosyula

Academic Editor

PLOS ONE

Dear Dr. Vallabhajosyula

We thank you and reviewers for the generous comments on the manuscript and have revised the manuscript to address these concerns. Here we enclose our point-by-point responses to the comments raised by the reviewers and editors. We hope our responses and revisions made to the manuscript can address these concerns. We are looking forward to your positive response.

Yours sincerely,

Zhongheng Zhang, MD

Department of Emergency Medicine,

Sir Run Run Shaw Hospital,

Zhejiang University School of Medicine,

Hangzhou,

310016,

China.

Email: [zh_zhang1984@zju.edu.cn](mailto:zh_zhang1984@zju.edu.cn)

Additional Editor Comments:

**REVIEWER COMMENT**: I agree with all the comments from the Reviewers. Specifically, Reviewer 3 who mentions overlap with two prior publications - This study with the different subphenotypes has been published earlier, the previous study which has been published in Nature is entitled: Subphenotypes of Cardiac Arrest Patients Admitted to Intensive Care Unit: a latent profile analysis of a large critical care database, link to the article is attached below: https://doi.org/10.1038/s41598-019-50178-0. Most of the information in the above mentioned study has been almost copied verbatim in the present study entitled: Stability of Subphenotypes of Cardiac Arrest Patients Admitted to Intensive Care Unit: a latent transition analysis of a large critical care database. The only additional information in this study in the cross over of subphenotypes and it's effect on mortality on day 3 of hospitalization.
I suggest the authors consider addressing this in great detail to distinguish it from existing papers.

**RESPONSE**: Many thanks for the constructive suggestions and we thoroughly revised the manuscript to avoid verbatim. Futhermore, we added some lines in the introduction section to describe our previous work and how can the present study add to the existing literature.

**RELATED REVISED MANUSCRIPT TEXT (or Table/Figure)**: Our previous work has identified subphenotypes of CA using cross-sectional data on the first day of ICU entry [21]. However, it is largely unknown whether the subphenotypes are stable or subject to transitions and how this transition can impact clinical outcomes. Other studies show that subphenotype transition can have significant clinical implications [17,22]. Thus, the current study aimed to characterize the latent transition pattern of CA patients by using latent transition analysis (LTA). The differences in the mortality outcome for patients with different transition paths were also explored.

**MANUSCRIPT LOCATION**: P3 L24

Reviewer #1:

**REVIEWER COMMENT**: The authors of the present study present a well thought out analysis of a secondary database to evaluate sub-phenotypes of cardiac arrest. The authors conclude that the study identified three sub-phenotypes of cardiac arrest which were consistent on day 1 and day 3 after ICU admission. What is unclear is the importance of the study findings. The three profiles identified by the authors (a baseline profile, one with neurologic injury and the third with multiorgan dysfunction) are not new and are recognized to be part of the pathology of post-cardiac arrest syndrome.
**RESPONSE**: We are grateful to the reviewer for this insightful comments. We agree with the reviewer for that the subphenotypes identified were consistent with the pathology of post-cardiac arrest syndrome. However, our study utilized big EHR data and analytics to support the pathology of post-cardiac arrest syndrome, making it more evidence based. Furthermore, we showed that these subphenotypes had important clinical implications for prognosis. We revised the manuscript in the discussion section to incorporate the important interpretation provided by the reviewer.

**RELATED REVISED MANUSCRIPT TEXT (or Table/Figure)**: The profiles of CA identified by the big data analytics are consistent with the pathology of post-cardiac arrest syndrome. The novelty of the study is that we further quantitatively described the epidemiology of the profiles as well as the transitions among these profiles based on a large critical care database. We also quantitatively show that patients with different transition trajectories present different clinical outcomes.

**MANUSCRIPT LOCATION**: P17 L15

**REVIEWER COMMENT**: It is well acknowledged that cardiac arrest may be followed by brain injury, systemic hypo-perfusion, and multiple organ dysfunction. Therefore, if anything the three profiles identified by the authors indicate patients at varying stages of disease post cardiac arrest. And this sequence of events entirely depends on the timing of arrest, management strategy and importantly underlying pathology.
**RESPONSE**: we fully agree with the reviewer for the constructive comments. We added possible reasons for CA in the revision according to co-existing diagnosis. In the database, there is no direct annotation to the cardiac arrest reasons; thus we also use the information of the type of ICU to deduce possible reasons for CA. we also added this point as a limitation in the revision.

Some drug interventions were also added to the table to implicate some difference in management between subtypes.

**RELATED REVISED MANUSCRIPT TEXT (or Table/Figure)**:

Table 3

| Variables | Total (n=848) | Profile 1 (n=632) | Profile 2 (n=116) | Profile 3 (n=100) | p | |
| --- | --- | --- | --- | --- | --- | --- |
| Etiology, n (%) |  |  |  |  | 0.008 | |
| ARF | 257 (30) | 175 (28) | 50 (43) | 32 (32) |  | |
| MI | 129 (15) | 99 (16) | 15 (13) | 15 (15) |  | |
| Others | 293 (35) | 234 (37) | 30 (26) | 29 (29) |  | |
| Sepsis | 144 (17) | 109 (17) | 19 (16) | 16 (16) |  | |
| Trauma | 25 ( 3) | 15 (2) | 2 (2) | 8 (8) |  | |
| Type of care unit, n(%) |  |  |  |  | 0.001 | |
| CCU | 265(31) | 212(34) | 35(30) | 18(18) |  | |
| CSRU | 145(17) | 102(16) | 17(15) | 26(26) |  | |
| MICU | 270(32) | 193(31) | 49(42) | 28(28) |  | |
| SICU | 87(10) | 63(10) | 11(9) | 13(13) |  | |
| TSICU | 81(10) | 62(10) | 4(3) | 15(15) |  | |
| Dopamine, n(%) | 161(19) | 114(18) | 19(16) | 28(28) | | 0.046 |
| Epinephrine, n(%) | 79(9) | 48(8) | 9(8) | 22(22) | | 0.000 |
| Norepinephrine, n(%) | 272(32) | 166(26) | 40(34) | 66(66) | | 0.000 |
| Dobutamine, n(%) | 37(4) | 28(4) | 1(1) | 8(8) | | 0.037 |

Abbreviations: CCU: coronary artery unit; CSRU: cardiac surgery recovery unit; MICU: medical ICU; SICU: surgical ICU; TSICU: Trauma-Neuro ICU; ARF: acute respiratory failure; MI: myocardial infarction.

First, it is well acknowledged that cardiac arrest may be followed by brain injury, systemic hypo-perfusion, and multiple organ dysfunction. However, there is no direct annotation for the reasons of CA in the database. Thus, we included coexisting diagnosis as possible reasons of the CA. Furthermore, possible reasons for CA can also be deduced by the type of ICU. For example, the possible reason of CA in CCU is most probably myocardial infarction or arrythmia, and MICU patients may suffer from multiple organ failure.

**MANUSCRIPT LOCATION**: Table 3. P19 L11.

**REVIEWER COMMENT**: Can the authors provide any information on the cause of cardiac arrest, timing of arrest along with if and how these patients were managed? Providing analytical results without clinical picture does not add anything to aid in decision making for these patients.
**RESPONSE**: we fully agree with the reviewer on this point that analytical results without clinical picture does not add anything to aid in decision making for these patients. However,there is no direct annotation of the reasons for CA events in the database. We added co-existing diagnosis as possible reasons for the CA. We also reported the type of ICU for possible reasons of CA. For example, the possible reason of CA in CCU is most probably myocardial infarction or arrythmia, and MICU patients may suffer from multiple organ failure. We added vasopressors and mechanical circulatory support as the intervention to the results as this information is well recorded in the database.

**RELATED REVISED MANUSCRIPT TEXT (or Table/Figure)**:

Table 3

| Variables | Total (n=848) | Profile 1 (n=632) | Profile 2 (n=116) | Profile 3 (n=100) | p | |
| --- | --- | --- | --- | --- | --- | --- |
| Etiology, n (%) |  |  |  |  | 0.008 | |
| ARF | 257 (30) | 175 (28) | 50 (43) | 32 (32) |  | |
| MI | 129 (15) | 99 (16) | 15 (13) | 15 (15) |  | |
| Others | 293 (35) | 234 (37) | 30 (26) | 29 (29) |  | |
| Sepsis | 144 (17) | 109 (17) | 19 (16) | 16 (16) |  | |
| Trauma | 25 ( 3) | 15 (2) | 2 (2) | 8 (8) |  | |
| Type of care unit, n(%) |  |  |  |  | 0.001 | |
| CCU | 265(31) | 212(34) | 35(30) | 18(18) |  | |
| CSRU | 145(17) | 102(16) | 17(15) | 26(26) |  | |
| MICU | 270(32) | 193(31) | 49(42) | 28(28) |  | |
| SICU | 87(10) | 63(10) | 11(9) | 13(13) |  | |
| TSICU | 81(10) | 62(10) | 4(3) | 15(15) |  | |
| Dopamine, n(%) | 161(19) | 114(18) | 19(16) | 28(28) | | 0.046 |
| Epinephrine, n(%) | 79(9) | 48(8) | 9(8) | 22(22) | | 0.000 |
| Norepinephrine, n(%) | 272(32) | 166(26) | 40(34) | 66(66) | | 0.000 |
| Dobutamine, n(%) | 37(4) | 28(4) | 1(1) | 8(8) | | 0.037 |
| MCS/ECMO, n (%) | 102 (12) | 77 (12) | 6 (5) | 19 (19) | | 0.008 |

Abbreviations: CCU: coronary artery unit; CSRU: cardiac surgery recovery unit; MICU: medical ICU; SICU: surgical ICU; TSICU: Trauma-Neuro ICU.

First, it is well acknowledged that cardiac arrest may be followed by brain injury, systemic hypo-perfusion, and multiple organ dysfunction. However, there is no direct annotation for the reasons of CA in the database. Thus, we included coexisting diagnosis as possible reasons of the CA. Furthermore, possible reasons for CA can also be deduced by the type of ICU. For example, the possible reason of CA in CCU is most probably myocardial infarction or arrythmia, and MICU patients may suffer from multiple organ failure.

**MANUSCRIPT LOCATION**: Table 3. P19 L11.

**REVIEWER COMMENT**: The authors go on to compare outcomes of the three identified profiles. As previously mentioned, it appears these patients are at different stages of the disease and without knowing granular information on the extent and causes of disease, these outcome comparisons are not of value.
**RESPONSE**: In line with previous comments, we added the type of ICU/etiology of CA and some drug/mechanical support interventions in the result section. A new section with the subtitle “Interventions for the three profiles of CA” was added in this round of revision.

**RELATED REVISED MANUSCRIPT TEXT (or Table/Figure)**:

## Interventions for the three profiles of CA

There were significant differences in the drug intervention across the three subtypes of CA. For example, profile 3 was more likely to use dopamine than profile 1 and 2. While profile 2 used more norepinephrine than profile 1, profile 1 used more dopamine than profile 2 (Table 3). Profile 3 was more likely to use circulatory support than profile 2 (19% vs. 5%; p = 0.008).

**MANUSCRIPT LOCATION**: Table 3; P11 L4

**REVIEWER COMMENT**: It is understandable that sicker patients respond and get better or worse depending on the timeliness, efficacy and other intrinsic factors of treatment strategies. Do we need an analysis to establish this especially with a dynamic condition like post-cardiac arrest pathology?
**RESPONSE**: We are sorry for not making the rationale of the study clear and straightforward in the first version. We added some lines in the introduction in this round of revision to clarify this point. The rationale for this study is described in the introduction.

**RELATED REVISED MANUSCRIPT TEXT (or Table/Figure)**: Our previous work has identified subphenotypes of CA using cross-sectional data on the first day of ICU entry [21]. However, it is largely unknown whether the subphenotypes are stable or subject to transitions and how this transition can impact clinical outcomes. Other studies show that subphenotype transition can have significant clinical implications [17,22]. Thus, the current study aimed to characterize the latent transition pattern of CA patients by using latent transition analysis (LTA). The differences in the mortality outcome for patients with different transition paths were also explored.

**MANUSCRIPT LOCATION**: P3 L24

**REVIEWER COMMENT**: A more clear presentation of the clinical profiles along with management of these patients may be of value to the reader.

**RESPONSE**: We fully agree with the reviewer in this point. In the revision, we added some more sections to show the medication and clinical difference between the clinical profiles.

**RELATED REVISED MANUSCRIPT TEXT (or Table/Figure)**:

Table 3

| Variables | Total (n=848) | Profile 1 (n=632) | Profile 2 (n=116) | Profile 3 (n=100) | p | |
| --- | --- | --- | --- | --- | --- | --- |
| Type of care unit, n(%) |  |  |  |  | 0.001 | |
| CCU | 265(31) | 212(34) | 35(30) | 18(18) |  | |
| CSRU | 145(17) | 102(16) | 17(15) | 26(26) |  | |
| MICU | 270(32) | 193(31) | 49(42) | 28(28) |  | |
| SICU | 87(10) | 63(10) | 11(9) | 13(13) |  | |
| TSICU | 81(10) | 62(10) | 4(3) | 15(15) |  | |
| Dopamine, n(%) | 161(19) | 114(18) | 19(16) | 28(28) | | 0.046 |
| Epinephrine, n(%) | 79(9) | 48(8) | 9(8) | 22(22) | | 0.000 |
| Norepinephrine, n(%) | 272(32) | 166(26) | 40(34) | 66(66) | | 0.000 |
| Dobutamine, n(%) | 37(4) | 28(4) | 1(1) | 8(8) | | 0.037 |
| MCS/ECMO, n (%) | 102 (12) | 77 (12) | 6 (5) | 19 (19) | | 0.008 |

Abbreviations: CCU: coronary artery unit; CSRU: cardiac surgery recovery unit; MICU: medical ICU; SICU: surgical ICU; TSICU: Trauma-Neuro ICU; MCS: mechanical circulatory support; ECMO: Extracorporeal membrane oxygenation.

## Interventions for the three profiles of CA

There were significant differences in the drug intervention across the three subtypes of CA. For example, profile 3 was more likely to use dopamine than profile 1 and 2. While profile 2 used more norepinephrine than profile 1, profile 1 used more dopamine than profile 2 (Table 3). Profile 3 was more likely to use circulatory support than profile 2 (19% vs. 5%; p = 0.008).

**MANUSCRIPT LOCATION**: Table 3; P11 L4

Reviewer #2:

**REVIEWER COMMENT**: I had the chance to review the manuscript “Stability of Subphenotypes of Cardiac Arrest Patients Admitted to Intensive Care Unit: a latent transition analysis of a large critical care database”. The manuscript is well-written, scientifically sound and targets an interesting topic. The authors do a great job in highlighting subgroups that may benefit from individualized management strategies. I have a few comments for the authors’ consideration.
**RESPONSE**: Thank you for the insightful comments. We have revised the manuscrupt with point-by-point responses to each question.

**RELATED REVISED MANUSCRIPT TEXT (or Table/Figure)**: None

**MANUSCRIPT LOCATION**: None

Major comments
**REVIEWER COMMENT**: 1. Inclusion of mechanical circulatory support (MCS) and ECMO as variables for the LTA and LPA would be extremely valuable. It would also be instructive for the readers if the use of MCS and ECMO could be included in Table-3.

**RESPONSE**: we fully agree with the reviewer on this point and added such information in the revision in table 3. However, we do not agree to include interventions into the LPA/LTA models because these models generally uses features/characteristics.

**RELATED REVISED MANUSCRIPT TEXT (or Table/Figure)**:

Table 3 Baseline characteristics and outcomes by profiles on day 1

| MCS/ECMO, n (%) | 102 (12) | 77 (12) | 6 (5) | 19 (19) | 0.008 |
| --- | --- | --- | --- | --- | --- |

Profile 3 was more likely to use circulatory support than profile 2 (19% vs. 5%; p = 0.008).

**MANUSCRIPT LOCATION**: Table 3; P11 L8.

**REVIEWER COMMENT**: 2. I would recommend using multiple ICD-9 codes for CA rather than just one code (427.5). ICD-9-CM codes identify CA with varying accuracy and outcomes of CA vary depending on the administrative definition used. (Please refer to Vallabhajosyula et al. Mayo Clin Proc. 2020 doi: 10.1016/j.mayocp.2019.12.007).

**RESPONSE**: we are grateful to the reviewer for this insightful comment. We are sorry for not making the inclusion criteria clear in the original version, we searched both ICD-9 code and diagnosis including cardiac arrest, cardiopulmonary resuscitation and ventricular fibrilation. Thus, although the ICD-9 code for CPR and VF were not used, but we have actually included such patients. We added this to the revision to ensure the inlcuded patients represented cardiac arrest target population.

**RELATED REVISED MANUSCRIPT TEXT (or Table/Figure)**: Subjects with the diagnosis of cardiopulmonary resuscitation (ICD-9 code: 9960 and 9963), cardiac arrest (ICD-9 code: 4275) and ventricular fibrillation (ICD-9 code: 4274) were screened for potential eligibility[25].

**MANUSCRIPT LOCATION**: P5 L15

**REVIEWER COMMENT**: 3. Include a section on the limitations of the study.

**RESPONSE**: we included a section for limitation in this round of revision.

**RELATED REVISED MANUSCRIPT TEXT (or Table/Figure)**: Several limitations of the study must be acknowledged. First, it is well acknowledged that cardiac arrest may be followed by brain injury, systemic hypo-perfusion, and multiple organ dysfunction. However, there is no direct annotation for the reasons of CA in the database. Thus, we included coexisting diagnosis as possible reasons of the CA. Furthermore, possible reasons for CA can also be deduced by the type of ICU. For example, the possible reason of CA in CCU is most probably myocardial infarction or arrythmia, and MICU patients may suffer from multiple organ failure. Second, the study failed to find any interventions that were associated with transition patterns. Most probably, the transition pattern is an intrinsic nature of the disease, and interventions such as vasopressors and circulatory support cannot change the transition pattern. Another reason is that the interventions being explored are limited, numerous interventions are being used during post-CA care. We cannot explore all of them in one study. Third, our study showed that patients who transitioned from profile 3 to profile 1 did not demonstrate an impact on mortality compared to those who remained in profile 3. This is counterintuitive from clinical expertise. The result showed a trend that patients remained in profile 3 had 3-fold increase in mortality but the statistical significance was not reached, which could be due to the small sample size in profile 3 in the study.

**MANUSCRIPT LOCATION**: P19 L11

**REVIEWER COMMENT**: 4. If the database allows, stratifying Cardiac arrest by the etiology would enrich the paper. The clinical course, management, and outcomes of CA secondary to different etiologies (ie. MI, Sepsis, ARDS etc ) are quite distinct and targeting individual pathologies would be of benefit.

**RESPONSE**: we fully agree with the reviewer on this point and added these etiology into the analysis in this round of revision.

**RELATED REVISED MANUSCRIPT TEXT (or Table/Figure)**:

| Etiology, n (%) |  |  |  |  | 0.008 |
| --- | --- | --- | --- | --- | --- |
| ARF | 257 (30) | 175 (28) | 50 (43) | 32 (32) |  |
| MI | 129 (15) | 99 (16) | 15 (13) | 15 (15) |  |
| Others | 293 (35) | 234 (37) | 30 (26) | 29 (29) |  |
| Sepsis | 144 (17) | 109 (17) | 19 (16) | 16 (16) |  |
| Trauma | 25 ( 3) | 15 (2) | 2 (2) | 8 (8) |  |

Abbreviations: ARF: acute respiratory failure; MI: myocardial infarction.

**MANUSCRIPT LOCATION**: Table 3

**REVIEWER COMMENT**: 5. In the discussion the authors mention that the transition process may reflect the treatment strategies used. Would it be possible to assess the different treatment strategies used in patients who transitioned from profile 2 and profile 3 to profile 1 compared to those who remained in their respective profiles?

**RESPONSE**: we are greateful to the reviewer for this valuable comments. In this revision, we performed more analysis on the differences in treatment strategy for patients with different transition pattern.

**RELATED REVISED MANUSCRIPT TEXT (or Table/Figure)**:

## Impact of therapeutic intervention on profile transition

The associations of medical interventions, such as vasoactive agents and circulatory support, with the transition pattern were explored by univariate analysis. We compared patients transitioned from profile 2 and 3 to profile 1 versus those did not transition to profile 1 (Table 5). The results showed that there was no significant difference between the two transition groups in terms of medications and mechanical circulatory support. Probably, the transition pattern is the intrinsic nature of the disease progression, there is no effective therapeutic intervention that could change the transition path.

Table 5. Comparisons between patients who transitioned from profile 2 or 3 to profile 1 versus those not transitioned to profile 1.

| Variables | Total (n = 216) | Not transition to profile 1 (n = 51) | Transition to profile 1 (n = 165) | p |
| --- | --- | --- | --- | --- |
| Age (years), Median (IQR) | 66.00 (54.12, 78.10) | 66.33 (55.26, 75.85) | 65.44 (54.07, 78.97) | 0.806 |
| Gender, Male (%) | 136 (63) | 33 (65) | 103 (62) | 0.897 |
| SOFA, Median (IQR) | 10.00 (7.00, 12.00) | 9.00 (7.00, 10.50) | 10.00 (8.00, 12.00) | 0.118 |
| Dopamine use, n (%) | 47 (22) | 7 (14) | 40 (24) | 0.162 |
| Epinephrine use, n (%) | 31 (14) | 7 (14) | 24 (15) | 1.000 |
| Norepinephrine use, n (%) | 106 (49) | 21 (41) | 85 (52) | 0.258 |
| Dobutamine use, n (%) | 9 ( 4) | 1 ( 2) | 8 ( 5) | 0.689 |
| MCS/ECMO, n (%) | 191 (88) | 45 (88) | 146 (88) | 1.000 |
| Etiology, n (%) |  |  |  | 0.777 |
| ARF | 82 (38) | 20 (39) | 62 (38) |  |
| MI | 30 (14) | 6 (12) | 24 (15) |  |
| Others | 59 (27) | 12 (24) | 47 (28) |  |
| Sepsis | 35 (16) | 11 (22) | 24 (15) |  |
| Trauma | 10 ( 5) | 2 ( 4) | 8 ( 5) |  |

Abbreviations: SOFA: sequential organ failure assessment; MCS: mechanical circulatory support; ECMO: Extracorporeal membrane oxygenation; ARF: acute respiratory failure; MI: myocardial infarction.

**MANUSCRIPT LOCATION**: P13 L16; Table 5.

**REVIEWER COMMENT**: 6. The authors discuss the impact of using the profiling of cardiac arrest patients for clinical trials. It would be informative for readers if they could discuss a proposed clinical approach / management strategy to the 3 distinct profiles encountered on day 1.

**RESPONSE**: We fully agree with the reviewer on this constructive suggestion. We added more analyses in this revision to explore potential beneficial effects of interventions for patients with different transition pattern, however, no intervention was found to be associted with the transition pattern. We acknowledge this limitation in the revision. Futhermore, we added more discussions on the use of different interventions on day 1.

**RELATED REVISED MANUSCRIPT TEXT (or Table/Figure)**:

For instance, clinical trials designed to investigate agents or interventions with neurological protective property should be performed in *profile 2*. In a recent study, Nishikimi M and colleagues showed that the effect of mild therapeutic hypothermia was different depending on the presence or absence of hypoxic encephalopathy [30]. The effects of organ support interventions such as mechanical circulatory support (MCS) and ECMO can be investigated in *profile 3* patients.

Second, the study failed to find any interventions that were associated with transition patterns. Most probably, the transition pattern is an intrinsic nature of the disease, and interventions such as vasopressors and circulatory support cannot change the transition pattern. Another reason is that the interventions being explored are limited, numerous interventions are being used during post-CA care. We cannot explore all of them in one study.

**MANUSCRIPT LOCATION**: P17 L24; P19 L17

Minor comments

**REVIEWER COMMENT**: 1. Consider discussing why the patients who transitioned from profile 3 to profile 1 did not demonstrate an impact on mortality compared to those who remained in profile 3.

**RESPONSE**: we added discussion on this point in the revision.

**RELATED REVISED MANUSCRIPT TEXT (or Table/Figure)**: Third, our study showed that patients who transitioned from profile 3 to profile 1 did not demonstrate an impact on mortality compared to those who remained in profile 3, which is counterintuitive as judged by clinical expertise. The result showed a trend that patients remained in profile 3 had 3-fold increase in mortality but the statistical significance was not reached, which could be explained by the small sample size in profile 3 in the study.

**MANUSCRIPT LOCATION**: P19 L21

**REVIEWER COMMENT**: 2. Line 6 in the Introduction section tends to suggest that TTM has only shown success only in animal studies and not human studies which is not accurate as several studies demonstrated that TTM has improved survival and neurological function in patients with CA. This could be rephrased. (Please refer to the following doi:10.1161/CIR.0000000000000313; doi:10.1056/NEJMoa012689; doi:10.1056/NEJMoa003289).

**RESPONSE**: we rephrased this text in the revision.

**RELATED REVISED MANUSCRIPT TEXT (or Table/Figure)**: In particular, targeted temperature management (TTM) has been shown to improve survival and neurological functions in patients with CA[9-11].

**MANUSCRIPT LOCATION**: P3 L8
**REVIEWER COMMENT**: 3. Page 18 Line 23. “In this study, we found that the profile 2 had significantly greater…” Shouldn’t it be profile 3.

**RESPONSE**: We are sorry for this mistake and we correct it in the revision.

**RELATED REVISED MANUSCRIPT TEXT (or Table/Figure)**: In the study, we found that the *profile 3* had significantly greater proportions of the patients on vasopressors and inotropes.

**MANUSCRIPT LOCATION**: P19 L2
**REVIEWER COMMENT**: 4. Line 9 of the discussion should be ‘transitioned to profile 1’ instead of ‘profile 3’.

**RESPONSE**: corrected

**RELATED REVISED MANUSCRIPT TEXT (or Table/Figure)**: A substantial number of patients in profile 2 (72%) and 3 (82%) on day 1 transitioned to profile 1

**MANUSCRIPT LOCATION**: P17 L9
**REVIEWER COMMENT**: 5. The last sentence of the first paragraph in the discussion section needs to be rephrased.

**RESPONSE**: corrected

**RELATED REVISED MANUSCRIPT TEXT (or Table/Figure)**: Not surprisingly, the mortality outcome was significantly better for patients transitioned to *profile 1* on day 3 than those transitioned or remained in *profile 2* or 3.

**MANUSCRIPT LOCATION**: P17 L13

Reviewer #3:

**REVIEWER COMMENT**: In this original manuscript entitled,” Stability of Subphenotypes of Cardiac Arrest Patients Admitted to Intensive Care Unit:
a latent transition analysis of a large critical care database”,. This is a retrospective US based critical care database study that classifies cardiac arrest into three subphenotypes using the latent transition analysis and evaluates the stability of the three subphenotypes and effects on the ICU mortality outcomes. 848 patients were included and the study demonstrates that patients who transitioned to subphenotype 3 on day 3 of hospitalization had worse survival outcomes.
**RESPONSE**: Thank you for the comments.

**RELATED REVISED MANUSCRIPT TEXT (or Table/Figure)**: None

**MANUSCRIPT LOCATION**: None
major comments
**REVIEWER COMMENT**: 1. The introduction and discussion can be more focused on the implications of the stability and transition of subphenotypes

**RESPONSE**: We revised the introduction to meet the requirement.

**RELATED REVISED MANUSCRIPT TEXT (or Table/Figure)**: In addition, the subphenotype transition has also been widely investigated because unraveling the transition pattern can have significant clinical and research implications[16-18]. For example, subphenotype stability over time can help to design trials and/or therapeutics. Subphenotype transition is also important to the question on whether difference in clinical presentation is dependent on the timing of measurement[16].

However, it is largely unknown whether the subphenotypes are stable or subject to transitions and how this transition can impact clinical outcomes. Other studies show that subphenotype transition can have significant clinical implications [15,16]. Thus, the current study aimed to characterize the latent transition pattern of CA patients by using latent transition analysis (LTA).

**MANUSCRIPT LOCATION**: P3 L17; P3 L25
**REVIEWER COMMENT**: 2. This study is similar to your previous study entitled ' Subphenotypes of Cardiac Arrest Patients Admitted to Intensive Care Unit: a latent profile analysis of a large critical care database. would recommend you mention the previous study and highlight the findings of the new study.

**RESPONSE**: we cited our previous work in the revision and added some sentences on how can the current study add new.

**RELATED REVISED MANUSCRIPT TEXT (or Table/Figure)**: Our previous work has identified subphenotypes of CA using cross-sectional data on the first day of ICU entry [14]. However, it is largely unknown whether the subphenotypes are stable or subject to transitions and how this transition can impact clinical outcomes. Other studies show that subphenotype transition can have significant clinical implications [15,16]. Thus, the current study aimed to characterize the latent transition pattern of CA patients by using latent transition analysis (LTA). The differences in the mortality outcome for patients with different transition paths were also explored.

**MANUSCRIPT LOCATION**: P3 L24
