# Supplementary material for: Latent transition analysis of cardiac arrest patients treated in the intensive care unit
Source: PLoS One. 2021 May 27;16(5):e0252318. doi: 10.1371/journal.pone.0252318 (PMC8158944; doi:10.1371/journal.pone.0252318)
Supplement: Attachment — Submitted filename: Response to Reviewers.docx [file pone.0252318.s002.docx]

To Dr. Saraschandra Vallabhajosyula

Academic Editor

PLOS ONE

Dear Dr. Vallabhajosyula

We thank you and reviewers for the generous comments on the manuscript and have revised the manuscript to address these concerns. Here we enclose our point-by-point responses to the comments raised by the reviewers and editors. We hope our responses and revisions made to the manuscript can address these concerns. We are looking forward to your positive response.

Yours sincerely,

Zhongheng Zhang, MD

Department of Emergency Medicine,

Sir Run Run Shaw Hospital,

Zhejiang University School of Medicine,

Hangzhou,

310016,

China.

Email: [zh_zhang1984@zju.edu.cn](mailto:zh_zhang1984@zju.edu.cn)

Reviewer #2: This paper titled “Latent transition analysis of cardiac arrest patients treated in the intensive care unit” focuses on sub-phenotypes of cardiac arrest patients on day 1 and day 3 of admission and the transition between the identified groups. The paper is scientifically sound and well written. I appreciate the efforts the authors made to address all the concerns raised by the reviewers. I would like to mention a few minor comments I have

**REVIEWER COMMENT**: 1. The manuscript would benefit from copyediting.

**RESPONSE**: the tables intersted in the main text were editted and some other places were also editted for clarity and typos.

**RELATED REVISED MANUSCRIPT TEXT (or Table/Figure)**: multiple places

**MANUSCRIPT LOCATION**: multiple places.

**REVIEWER COMMENT**: 2. Although the overlap with your prior manuscript has been addressed, attempts to minimize this further would be appreciated.

**RESPONSE**: the manuscript was revised futher to reduce overlap.

**RELATED REVISED MANUSCRIPT TEXT (or Table/Figure)**: multiple places

**MANUSCRIPT LOCATION**: multiple places.

**REVIEWER COMMENT**: 3. Rephrase Page 19 line 19, 20 and page 14 line 5 – The fact that there was no difference in the medications between the patients that transitioned vs those that did not transition could reflect an intrinsic nature of the disease and lack of identifiable interventions that were associated with transition. However, concluding that the interventions cannot change transition might not be appropriate as patients requiring vasopressors/ inotrope/ TTM/ circulatory support and not receiving it will surely lead to deleterious effects.

**RESPONSE**: we fully agree with the reviewer on this point and revised the texts.

**RELATED REVISED MANUSCRIPT TEXT (or Table/Figure)**: Second, the study failed to find any interventions that were associated with transition patterns. Most probably, the fact that there was no difference in the medications between the patients that transitioned versus those that did not transition could reflect an intrinsic nature of the disease and lack of identifiable interventions that were associated with transitions. Other reasons are that the number of interventions being explored is limited and the study may not have enough power to detect some small effect sizes.

**MANUSCRIPT LOCATION**: P19 L15

Reviewer #3: I had the opportunity to re-review the manuscript entitled " Latent transition analysis of cardiac arrest patients treated in the intensive care unit".
**REVIEWER COMMENT**: 1.Most of the revisions that have been suggested by me and the other reviewer's and editor has been incorporated in the manuscript.

**RESPONSE**: Thank you for the comments.

**REVIEWER COMMENT**: 2. The manuscript has been revised to not mirror it's previous study verbatim entitled ' Subphenotypes of Cardiac Arrest Patients Admitted to Intensive Care Unit: a latent profile analysis of a large critical care database'.

**RESPONSE**: Thank you for the comments.

**REVIEWER COMMENT**: 3. The sub-phenotypes of CA indicate a continuum of the disease process of CA and post cardiac arrest syndrome, the manuscript is not clearly demonstrating the clinical implications (not the further research implications) of the sub-phenotypes. The future research implication of the transition of the sub-phenotypes have been discussed in detail in this revision of the manuscript.

**RESPONSE**: we add some lines to discuss the clinical implications of the sub-phenotypes.

**RELATED REVISED MANUSCRIPT TEXT (or Table/Figure)**:

The transition pattern of post-cardiac arrest syndrome is helpful for risk stratification, which is important for medical resource allocation and decision making. Furthermore, transition pattern might be indicative of medical interventions that can help to direct treatment, although current study failed to identify any difference in interventions across different transition patterns.

**MANUSCRIPT LOCATION**: P17 L20

**REVIEWER COMMENT**: Furthermore, There is a statement that is contradicting the conclusions and the clinical implications aspect of the discussion. The authors themselves state that ' Probably, the transition pattern is the intrinsic nature of the disease progression, there is no effective therapeutic intervention that could change the transition path' . but further go on to discuss the possible interventions that could be carried out at different sub-phenotype profile. This statement has to be modified or addressed in the discussion as it is contradicting the study aim.

**RESPONSE**: The statement has been modified in the revision.

**RELATED REVISED MANUSCRIPT TEXT (or Table/Figure)**:

Second, the study failed to find any interventions that were associated with transition patterns. Most probably, the fact that there was no difference in the medications between the patients that transitioned versus those that did not transition could reflect an intrinsic nature of the disease and lack of identifiable interventions that were associated with transitions. Other reasons are that the number of interventions being explored is limited and the study may not have enough power to detect some small effect sizes.

**MANUSCRIPT LOCATION**: P19 L15
